# Genetic architecture of lipid traits in the Hispanic community health study/study of Latinos

**DOI:** 10.1186/s12944-017-0591-6

**Published:** 2017-10-12

**Authors:** Mariaelisa Graff, Leslie S. Emery, Anne E. Justice, Esteban Parra, Jennifer E. Below, Nicholette D. Palmer, Chuan Gao, Qing Duan, Adan Valladares-Salgado, Miguel Cruz, Alanna C. Morrison, Eric Boerwinkle, Eric A. Whitsel, Charles Kooperberg, Alex Reiner, Yun Li, Carlos Jose Rodriguez, Gregory A. Talavera, Carl D. Langefeld, Lynne E. Wagenknecht, Jill M. Norris, Kent D. Taylor, George Papanicolaou, Eimear Kenny, Ruth J. F. Loos, Yii-Der Ida Chen, Cathy Laurie, Tamar Sofer, Kari E. North

**Affiliations:** 1 Department of Epidemiology, Gillings School of Global Public Health, University of North Carolina at Chapel Hill, Chapel Hill, NC 27599 USA; 2 Department of Biostatistics, University of Washington, Seattle, WA USA; 3 Biomedical and Translational Informatics, Geisinger Health, Danville, PA USA; 4 Department of Anthropology, University of Toronto at Mississauga, Mississauga, ON Canada; 5 Vanderbilt Genetics Institute, Vanderbuilt University, Nashville, TN USA; 6 Department of Biochemistry, Wake Forest University School of Medicine, Winston-Salem, NC USA; 7 Molecular Genetics and Genomics Program, Wake Forest University School of Medicine, Winston-Salem, NC USA; 8 Department of Genetics, University of North Carolina at Chapel Hill, Chapel Hill, NC USA; 9 Unidad de Investigacion Medica en Bioquimica, Hospital de Especialidades, CMNSXX1-IMSS, Mexico City, Mexico; 10 Department of Epidemiology, Human Genetics and Environmental Sciences, University of Texas Health Science Center at Houston, Houston, TX USA; 11 Department of Medicine, University of North Carolina at Chapel Hill, Chapel Hill, NC USA; 12 Fred Hutchinson Cancer Research Center, Public Health Sciences, Seattle, WA USA; 13 Department of Medicine and Public Health Sciences, Wake Forest University School of Medicine, Winston-Salem, NC USA; 14 Graduate School of Public Health, San Diego State University, San Diego, CA USA; 15 Department of Biostatistical Sciences, Wake Forest University School of Medicine, Winston-Salem, NC USA; 16 Department of Epidemiology, Colorado School of Public Health, University of Colorado Denver, Aurora, CO USA; 17 Institute for Translational Genomics and Population Sciences and Department of Pediatrics, Los Angeles BioMedical Research Institute at Harbor-UCLA Medical Center, Torrance, CA USA; 18 National Heart, Lung, and Blood Institute, Bethesda, MD 20892 USA; 19 Genetics and Genomic Sciences, Icahn School of Medicine at Mount Sinai, New York, NY USA; 20 The Charles Bronfman Institute for Personalized Medicine, Icahn School of Medicine at Mount Sinai, New York, NY USA; 21 Division of Sleep and Circadian Disorders, Brigham and Women’s Hospital, Boston, MA USA; 22 Department of Medicine, Harvard Medical School, Boston, MA USA

**Keywords:** HDL, LDL, Triglycerides, Cholesterol, Genetics, Ancestry, Hispanic/Latino

## Abstract

**Background:**

Despite ethnic disparities in lipid profiles, there are few genome-wide association studies investigating genetic variation of lipids in non-European ancestry populations. In this study, we present findings from genetic association analyses for total cholesterol, low density lipoprotein cholesterol (LDL), high density lipoprotein cholesterol (HDL), and triglycerides in a large Hispanic/Latino cohort in the U.S., the Hispanic Community Health Study / Study of Latinos (HCHS/SOL).

**Methods:**

We estimated a heritability of approximately 20% for each lipid trait, similar to previous estimates in Europeans. To search for novel lipid loci, we performed conditional association analysis in which the statistical model was adjusted for previously reported SNPs associated with any of the four lipid traits. SNPs that remained genome-wide significant (*P* < 5 × 10^−8^) after conditioning on known loci were evaluated for replication.

**Results:**

We identified eight potentially novel lipid signals with minor allele frequencies <1%, none of which replicated. We tested previously reported SNP-trait associations for generalization to Hispanics/Latinos via a statistical framework. The generalization analysis revealed that approximately 50% of previously established lipid variants generalize to HCHS/SOL based on directional FDR r-value < 0.05. Some failures to generalize were due to lack of power.

**Conclusions:**

These results demonstrate that many loci associated with lipid levels are shared across populations.

**Electronic supplementary material:**

The online version of this article (10.1186/s12944-017-0591-6) contains supplementary material, which is available to authorized users.

## Background

Lipid level profiles are important determinants of heart and vascular health [1], with a disproportionate prevalence of unhealthy lipid profiles among U.S. minority populations, especially Hispanics/Latinos [2, 3]. The most commonly studied lipid traits include total cholesterol, high-density lipoprotein cholesterol (HDL), low-density lipoprotein cholesterol (LDL), and triglycerides. High levels of LDL and triglycerides are considered risk factors for coronary heart disease (CHD) while the opposite is true for HDL levels [4] .

The estimated heritability of lipid traits from twin and family studies, generally studied in European descent populations, ranges from 20% to higher than 70% [5]. The largest genome-wide association study (GWAS) reported to date identified 157 common genetic variants (minor allele frequency [MAF] > 2%) in over 188,000 participants from European-descent populations [6, 7] and several of the loci marked by these variants also have secondary signals, defined as associations that remain (or become) statistically significant after conditioning on the most significant SNP in the region [8]. Collectively, these common variants explain ~30-33% of the phenotypic variance for these traits in samples of primarily European ancestry [8].

Despite the disparities surrounding unhealthy lipid profiles [2, 3], there is a dearth of genetic studies investigating lipids in non-European populations. While most lipid-associated loci were discovered in studies of Europeans, a few studies of lipids have identified population-specific signals in African, Asian, and Hispanic/Latino descent populations [9–15]. Previous studies [14] show that by studying multiple ethnicities one can leverage differences in associations, allele frequencies, and linkage disequilibrium (LD) patterns to fine-map known loci and narrow down the region in which functional variants are expected. Therefore, studies in diverse ethnicities are important to increase understanding of the differences in lipid profiles across populations, to ascertain whether these differences are due to genetic architecture, and finally, for fine-mapping of known loci.

In this study, we present findings from a large Hispanic/Latino cohort, the Hispanic Community Health Study / Study of Latinos (HCHS/SOL). We tested the association of more than 25 million genotyped or imputed variants with four lipid traits: LDL, HDL, total cholesterol, and triglycerides. Further, we sought to identify new signals within known association regions by implementing statistical models conditioned on previously reported associated SNPs [9, 11–14]. We assessed SNP associations that were significant in the conditional model for replication in a Hispanic/Latino meta-analysis, and for generalization in European- and African-descent study populations. In addition, we assessed the phenotypic variance in lipid traits explained by common SNPs across the genome in the HCHS/SOL.

## Methods

### Participants and study design

The HCHS/SOL is a community-based cohort study of 16,415 self-identified Hispanic/Latino individuals aged 18 - 74 from randomly selected households near four U.S. field sites (Chicago, IL; Miami, FL; Bronx, NY; and San Diego, CA) [16]. The two-stage probability sample design was previously described in LaVange et al. [17]. HCHS/SOL cohort includes participants who self-identified as having Hispanic/Latino background, the largest groups being Central American (*n* = 1732), Cuban (*n* = 2348), Dominican (*n* = 1473), Mexican (*n* = 6472), Puerto-Rican (*n* = 2728), and South American (*n* = 1072). Baseline lipids levels were measured for participants during clinical examinations from 2008 to 2011. Of the study population, 12,803 individuals both consented for genotyping and were successfully genotyped. The HCHS/SOL was approved by institutional review boards at participating institutions, and written informed consent was obtained from all participants.

### Genotyping and imputation

DNA extracted from whole blood was genotyped on an Illumina custom array consisting of the Illumina Omni 2.5 M array (HumanOmni2.5-8v1-1) and ~150,000 custom SNPs identified to capture Amerindian genetic variation. Sample-level quality and identity checks, and SNP-level quality filtering resulted in a total of 12,803 samples with a missing call rate < 1% and 2,232,944 informative SNPs with a missing call rate < 2%. Imputation with the 1000 Genomes Project phase 1 multi-ethnic reference panel using SHAPEIT2 pre-phasing and IMPUTE2 imputation resulted in 25,568,744 imputed variants. Genotype quality control, imputation, relatedness, and PC estimation methods are described in more detail in Conomos et al., 2016 [18].

### Lipids phenotype outcomes

Twelve-hour fasting blood samples were collected according to standard protocols and used to measure serum total cholesterol, triglycerides, and HDL levels [19]. LDL levels were estimated according to the Friedewald equation [20]. An inventory of all prescription and over-the-counter medication each participant had used in the previous four weeks was taken at the clinic examination. We retained individuals taking lipid-lowering medications, first because these individuals are likely to carry variants that result in dyslipidemia, and second, to maximize the sample size. Therefore, we adjusted lipid levels by adding constant values for participants who reported taking lipid-lowering medications (statins, fibrates, bile acid sequestrants, niacin, and cholesterol absorption inhibitors) as has been done previously [15]. This adjustment was made in an attempt to restore the lipid value to what it was before taking the medications. The constant value depended on the specific type of medications used (Additional file 1: Table S1) [4, 21, 22]. If multiple medications were used, we applied the correction factor with the largest effect (e.g. for someone on statins and fibrates, we adjusted their triglycerides level by +57.1 mg/dL and their LDL by +49.9 mg/dL). To assess potential biases from applying a medication correction, we performed sensitivity analyses with known lipid loci, where we included all individuals and 1) applied a correction factor by adding constant values for participants who reported taking lipid-lowering medications, or 2) adjusted for lipid-lowering medications using a separate covariate for each lipid drug. Results varied little regardless of how we accounted for users of lipid-lowering medication (Additional file 2: Fig. S1). One extreme triglyceride level was excluded from analysis (adjusted triglycerides = 6366 mg/dL). Triglyceride levels were log-transformed (after medication adjustment) for association analyses. All other lipid traits were normally distributed.

### Genetic association analyses

Tests for genetic associations were performed using linear mixed models (LMMs), adjusting for population structure using the first five genetic principal components (PCs) and “genetic analysis group” as fixed effects. Genetic analysis groups were defined from a combination of genetic PCs and self-identified ancestry [18]. We adjusted for sampling design using a function (determined by AIC) of the sampling weights as a fixed effect, and adjusted for correlation among individuals due to shared community (group block), household, and genetic relatedness (kinship) using random effects. Expected allelic dosages were used for imputed SNPs in the association analyses, and results were filtered according to the effective minor allele count accounting for imputation quality. A detailed description of genetic association analyses in the HCHS/SOL can be found in Conomos et al., 2016 [18]. Significance was assessed using a genome-wide significance threshold of *p*-value ≤5 × 10^−8^. A significant locus was defined as a 1 Mb region (+/−500 kb) around the most significant SNP (index SNP).

### Conditional analyses

To evaluate the possibility of novel lipid loci within previously established association regions, we applied the same LMMs for association testing while conditioning on previously reported index SNPs associated with any of the four lipids traits [6–15] (i.e. by including them as covariates), under the assumption of pleiotropy (Additional file 1: Table S2). In this table a ‘primary’ SNP was defined as the first identified (published) SNP in a given 1 Mb (+/− 500 kb) region, and an established ‘secondary’ SNP was defined as any published SNPs inside of a ‘primary’ SNP region. All primary SNPs are independent from each other and from all secondary SNPs, but not all secondary SNPs are independent from each other in a given region. SNPs that remained or became genome-wide significant after conditioning on known loci were considered for replication testing in other cohorts. In the results presented here, we defined potentially novel primary signals as previously-unreported SNPs that fell outside of a known index SNP region. We defined potentially novel secondary signals as previously-unreported if the SNP fell inside of a known index SNP region but was independent of all other SNPs within that region.

### Replication of potential novel signals

Eight SNPs that reached genome-wide significance, separate from previously reported signals, were tested for replication in independent studies. These consisted of samples from Mexican Americans from Starr County, Texas and individuals from Mexico City [9], women in the Women’s Health Initiative Study (WHI) who self-reported Hispanic/Latino ancestry [23], and a subset of cohorts from the GUARDIAN consortium consisting of participants of Mexican ancestry [Insulin Resistance Atherosclerosis Study [24], Insulin Resistance Atherosclerosis Family Study (IRAS-FS) [25], Hypertension-Insulin Resistance (HTN-IR) Family study [26], and Mexican-American Coronary Artery Disease (MACAD) study] [27]. We also sought to generalize novel association signals to populations of European or African ancestry, including individuals in the Atherosclerosis Risk in Communities (ARIC) study [28] and the Women’s Health Initiative Study (WHI) [23]. We selected individuals of different ancestries because many of the SNPs followed-up for replication testing were rare in the HCHS/SOL, but slightly more frequent in other ancestries based on reference samples including African (AFR) or European (EUR) individuals.

In each replication study, fasting lipid levels were collected using standardized procedures and lipid phenotypes were adjusted for medication use in a manner similar to the HCHS/SOL analyses, except for GUARDIAN who did not adjust for lipid medication due to <5% of use within each study. Each study used linear regression stratified by ancestry (i.e. European, African, and Hispanic/Latino) to test for SNP-trait associations while adjusting for covariates including age, sex, and PCs 1-10, and obtained *p*-values from the Wald test. Family-based cohorts adjusted for pedigree structure. We then performed both ancestry-specific and a combined ancestries inverse normal fixed effects meta-analyses. To test the hypothesis of an association in the replication studies, we used the framework of Sofer et al. [29] and calculated a false discovery rate (FDR)-controlling directional r-value for each tested association, based on its p-value in both the HCHS/SOL and the replication study. FDR was controlled at the 0.05 level in calculating the r-values in each replication analysis, and we concluded that an association replicated in a given follow-up meta-analysis if it had an r-value ≤0.05.

### Heritability estimation

To estimate the heritability of each lipid trait in the HCHS/SOL sample, we estimated kinship coefficients within a maximal set of unrelated participants, using the complete set of genotyped SNPs with minor allele frequencies (MAF) ≥1% (~1.7 million SNPs). Unrelated individuals were defined as those with estimated kinship coefficients smaller than 2^-11/2^, i.e. more distant than fourth degree relatives. We estimated heritability as the proportion of the total phenotypic variance explained by the kinship coefficient matrix, calculated in a LMM as described above. The LMM was adjusted for the same fixed and random effects as described above in genetic association analyses, with the exception that a slightly different kinship matrix was used. Heritability *p*-values were calculated based on the likelihood ratio test, and 95% confidence intervals based on the normal approximation to the distribution of the ratio between the kinship variance components and the total variance.

### Generalization of previously reported SNP-trait associations

We identified nine studies that previously reported SNP-lipid trait associations in cohorts of European ancestry [5–7], and other ancestries [8–13]. We investigated whether the 347 SNP-trait associations (some SNPs overlap with more than one lipid trait, Additional file 1: Table S2) reported in these studies generalized to Hispanics/Latinos in the HCHS/SOL sample. For each known lipid-associated SNP, we calculated an FDR-controlling directional r-value based on both the *p*-value reported in the literature, and the HCHS/SOL association testing results. We computed r-values for each study (i.e. the study in the literature and the HCHS/SOL study) and trait separately. An association was generalized if its corresponding r-value was smaller than 0.05.

For each lipid trait, we also computed a genetic risk score for the SNPs that did not generalize to determine the importance of power on negative results. Specifically, for each trait, we summed the trait-increasing alleles of all the non-generalized SNPs, and tested the resulting risk score in the linear mixed model described above. A p-value <0.05 indicates that, while not formally generalized, some of the SNPs are likely associated with the trait in Hispanics/Latinos.

## Results

The sample included participants (~59% female) who were on average 46.1 years of age, ranging from 18 to 74 years (Table 1). Approximately 12.3% of the individuals reported using lipid-lowering medications. Mean measured total cholesterol, LDL, HDL, and triglycerides were 199.49 (±43.6) mg/dl, 122.85 (±36.54) mg/dl, 49.07 (±13.09), and 139.75 (±101.03) mg/dl, respectively.

**Table 1:** Descriptives of analytic sample for each lipid trait

	HDL	LDL	Triglycerides	Total Cholesterol
Sample size, N	12,730	12,467	12,730	12,731
% Female	58.93	59.46	58.93	58.93
Mean age, years	46.1 (13.8)	46.1 (13.8)	46.1 (13.8)	46.1 (13.8)
% reporting use of lipid-lowering medications	12.27	12.25	12.26	12.27
Mean (SD) unadjusted lipid value (mg/dL)	49.07 (±13.09)	122.85 (±36.54)	139.75 (±101.03)	199.49 (±43.6)
Mean (SD) lipid value (mg/dL), adjusted for medication use^a^	48.73 (±13.17)	128.87 (±38.13)	142.6 (±102.34)	205.78 (±45.66)
Natural log of adjusted lipid value (mg/dL)^b^			4.79 (±0.56)	

^a^ A constant was added to the lipid value based on type medication use. See Additional file 1: Table S1

^b^ Only triglycerides were natural log transformed. Lipid values were corrected for medication use prior to natural log transformation

### Trait-specific association analyses

The genomic inflation factors for the association analyses of HDL, LDL, and total cholesterol were each 1.03 and for triglycerides was 1.00 (Additional file 2: Fig. S2), indicating adequate control of population stratification. In these trait-specific analyses, we identified 14, 16, 17, and 10 genome-wide significant independent loci (+/− 500 kb from index SNP) associated with HDL, LDL, total cholesterol, and triglycerides, respectively (Additional file 1: Table S3). We tested these loci for novelty as follows.

### Conditional analyses

To identify potentially novel signals, we conditioned the trait-specific association analyses on 344 previously identified variants for the four lipid traits in European, Asian, African, and Hispanic/Latino samples. We observed eight new independent genome-wide significant association signals in total; four for HDL, one for LDL, two for total cholesterol, and one for triglycerides (Additional file 1: Table S4).

Two of the four HDL signals were potentially novel secondary signals as they fell within +/− 500 kb of a known locus, one in *APOA5/A1* (rs184637772, MAF = 0.002) and one in *DAGLB (*rs77071750, MAF = 0.003). The additional six signals were potentially novel primary signals, as they fell outside +/− 500 kb of known loci: two signals associated with HDL, one in *SYNE1* (rs78768891, MAF = 0.007) and one in *AUTS2* (rs191891263, MAF = 0.003); one signal associated with LDL located near *DNAL1* (rs149886784, MAF = 0.002); one signal associated with triglycerides near *SMOC2* (rs77635931, MAF = 0.002); and two signals associated with total cholesterol, in *CD86* (rs114378860, MAF = 0.007), and near *DNAH5* (rs183336356, MAF = 0.002).

### Replication of potential novel signals

**Fig. 1:**
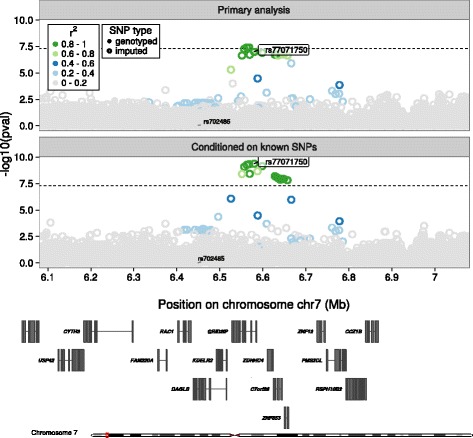
LocusZoom plots of the novel signal identified and replicated in the *DAGLB* locus (index variant rs77071750) that is independent of the known signal at rs702485. The top half of the figure “Primary analysis” shows the -log10 p-values for all variants before conditioning on the known variant. The bottom half of the figure “Conditioned on known SNPs” shows the -log10 p-values for all variants after conditioning on the known variant, rs702485

These potentially novel signals failed to replicate in our combined replication samples from GUARDIAN, the ARIC study, and WHI study, and an existing Mexican ancestry meta-analysis (Table 2). They also failed to replicate when we tested them in each separate meta-analyzed ancestry of the replication samples (i.e. European only, African only, and Hispanic only). Our power to replicate an effect in for each signal based on the replication meta-analyzed sample was less than 80% power except for the signal in *DAGLB* (rs77071750), which was 94%. This was partially from the larger replication sample available for thes signal and slightly higher MAF for in the combined replication sample, 0.08% versus 0.02% for HCHS/SOL. The signal in *DAGLB* (rs77071750) nearly reached significance in the combined meta-analyzed sample at r-value = 0.08. Figure 1 shows this signal before and after conditional analyses on the known SNP, rs702485.

**Table 2:** Replication and overall meta-analysis of 8 variants of interest with the indicated lipid trait

Analysis or Study^a^	rsID; Chr:Position; Effect/Other alleles ^b^	*Nearest Gene*	Sample size	Effect allele frequency	Beta	Standard error	Pvalue^c^	Meta-analysis generalization r-value^d^
Trait = Total Cholesterol								
Discovery study: HCHS/SOL	rs114378860; chr3:121,815,682; T/A	*CD86*	12,731	0.993	−17.930	3.091	6.62E-09	**1.00**
Replication meta-analysis, without HCHS/SOL			**27,334**	**0.984**	**0.013**	**0.058**	**8.20E-01**	
Replication study: GUARDIAN-MACAD Mexican Americans			726	0.991	0.001	0.060	9.84E-01	
Replication study: GUARDIAN-IRASFS Mexican Americans			1020	0.995	1.190	11.552	9.18E-01	
Replication study: Mexican Ancestry Samples^e^			3038	0.996	0.267	0.258	3.02E-01	
Replication study: WHI Hispanic Americans			3361	0.996	10.724	10.374	3.01E-01	
Replication study: ARIC African Americans			2488	0.966	−6.923	3.921	7.75E-02	
Replication study: WHI African Americans			7943	0.962	0.114	2.077	9.56E-01	
Replication study: ARIC European Americans			8758	0.999	−29.479	15.368	5.51E-02	
Overall meta-analysis with HCHS/SOL			40,065	0.987	0.007	0.058	9.06E-01	
Discovery study: HCHS/SOL	rs183336356; chr5:13,967,131; C/T	*22 kb 5′ of DNAH5*	12,731	0.998	−33.910	6.602	2.80E-07	
Replication meta-analysis, without HCHS/SOL			**13,792**	**0.991**	**−4.176**	**3.173**	**1.88E-01**	**0.63**
Replication study: WHI Hispanic Americans			3361	0.999	−5.673	15.464	7.14E-01	
Replication study: ARIC African Americans			2488	0.990	10.379	6.847	1.30E-01	
Replication study: WHI African Americans			7943	0.989	−8.296	3.680	2.42E-02	
Overall meta-analysis with HCHS/SOL			26,523	0.995	−9.755	2.860	6.47E-04	
Trait = Triglycerides								
Discovery study: HCHS/SOL	rs77635931; chr6:168,806,515: G/A	*RP1-39 J2.1;SMOC2*	12,730	0.998	−0.352	0.068	2.54E-07	**1.00**
Replication meta-analysis, without HCHS/SOL			**35,236**	**0.993**	**0.039**	**0.027**	**1.47E-01**	
Replication study: GUARDIAN-HTN Mexican Americans			732	0.996	−0.046	0.132	7.26E-01	
Replication study: Mexican Ancestry Samples			3047	0.998	0.284	0.355	4.24E-01	
Replication study: WHI Hispanic Americans			3361	0.997	0.256	0.126	4.19E-02	
Replication study: ARIC African Americans			2490	0.998	0.275	0.203	1.76E-01	
Replication study: WHI African Americans			7943	0.998	−0.161	0.094	8.72E-02	
Replication study: ARIC European Americans			8758	0.989	0.029	0.043	5.06E-01	
Replication study: WHI European Americans			8905	0.989	0.061	0.041	1.38E-01	
Overall meta-analysis with HCHS/SOL			47,966	0.995	−0.014	0.025	5.78E-01	
Trait = HDL Cholesterol								
Discovery study: HCHS/SOL	rs78768981; chr6:152,722,091; G/A	*SYNE1*	12,730	0.993	−5.522	0.962	9.29E-09	**0.63**
Replication meta-analysis, without HCHS/SOL			**16,841**	**0.977**	**−0.296**	**0.250**	**2.35E-01**	
Replication study: Mexican Ancestry Samples			3047	0.998	−0.197	0.290	4.97E-01	
Replication study: WHI Hispanic Americans			3361	0.996	5.007	2.504	4.55E-02	
Replication study: ARIC African Americans			2490	0.954	−1.852	1.168	1.13E-01	
Replication study: WHI African Americans			7943	0.954	−0.572	0.556	3.04E-01	
Overall meta-analysis with HCHS/SOL			29,571	0.984	−0.626	0.242	9.59E-03	
Discovery study: HCHS/SOL	rs77071750; chr7:6,579,655; C/G	*DAGLB/GRID2IP*	12,730	0.997	−7.975	1.498	1.01E-07	
Replication meta-analysis, without HCHS/SOL			22,551	0.992	−2.466	0.952	9.58E-03	0.08
Replication study: WHI Hispanic Americans			3361	0.998	4.821	5.864	4.11E-01	
Replication study: ARIC African Americans			2490	0.984	−2.364	2.221	2.87E-01	
Replication study: WHI African Americans			7943	0.985	−2.598	1.081	1.62E-02	
Replication study: ARIC European Americans			8757	1.000	−9.786	7.830	2.11E-01	
Overall meta-analysis with HCHS/SOL			35,281	0.994	−4.050	0.803	4.62E-07	
Discovery study: HCHS/SOL	rs191891263; chr7:69,496,422; A/G	*AUTS2*	12,730	0.998	−14.111	2.106	2.06E-11	
Replication meta-analysis, without HCHS/SOL			**15,074**	**0.984**	**0.083**	**0.415**	**8.41E-01**	**1.00**
Replication study: Mexican Ancestry Samples			1281	0.998	0.016	0.483	9.73E-01	
Replication study: WHI Hispanic Americans			3361	0.998	3.843	3.950	3.31E-01	
Replication study: ARIC African Americans			2489	0.983	3.863	2.238	8.44E-02	
Replication study: WHI African Americans			7943	0.977	−0.482	0.893	5.90E-01	
Overall meta-analysis with HCHS/SOL			27,804	0.991	−0.448	0.407	2.72E-01	
Discovery study: HCHS/SOL	rs184637772; chr11:116,702,251; T/C	*APOC3*	12,729	0.998	−7.698	1.637	2.57E-06	
Replication meta-analysis, without HCHS/SOL			13,794	0.995	2.468	1.303	5.82E-02	1.00
Replication study: WHI Hispanic Americans			3361	0.999	5.805	5.925	3.27E-01	
Replication study: ARIC African Americans			2490	0.993	4.410	3.143	1.61E-01	
Replication study: WHI African Americans			7943	0.993	1.833	1.475	2.14E-01	
Overall meta-analysis with HCHS/SOL			26,523	0.996	−1.474	1.019	1.48E-01	
Trait = LDL Cholesterol								
Discovery study: HCHS/SOL	rs149886784; chr14:74,138,147; C/T	*DNAL1*	12,467	0.998	−29.922	5.229	1.05E-08	**0.66**
Replication meta-analysis, without HCHS/SOL			**14,782**	**0.989**	**−2.612**	**2.692**	**3.32E-01**	
Replication study: GUARDIAN-IRASFS Mexican Americans			1007	0.995	−1.584	10.112	8.76E-01	
Replication study: WHI Hispanic Americans			3359	0.998	−3.031	11.855	7.98E-01	
Replication study: ARIC African Americans			2474	0.988	−9.717	6.136	1.13E-01	
Replication study: WHI African Americans			7942	0.985	−0.690	3.253	8.32E-01	
Overall meta-analysis with HCHS/SOL			27,249	0.993	−8.334	2.394	4.98E-04	

^a^ Replication studies included when the SNP was available and also met a minor allele count of ≥3. The results from the replication meta-analysis for each SNP is bolded

^b^ rsID according to dbSNP; Chromosome and Position are based on Human Genome Assembly 19

^c^ P-values are from unconditioned analyses

^d^ The directional FDR r-value for a SNP is the lowest directional FDR level at which we can say that it is generalized (replicated) with the same direction of association in both the discovery and generalizing studies. FDR r-values for generalization are based on one-side *P*-values with significance set at r-values <0.05

^e^ Mexican Ancestry samples come from the cohort of Starr County Mexican Americans and the Mexico City sample (Below et al. 2016) [9]

### Heritability estimates of lipid traits

SNP-based heritability was estimated from a variance-component analysis performed with a subset of 10,264 individuals that excluded close familial relatives. The estimated heritability was 22% for total cholesterol, 24% for triglycerides, 24% for HDL, and 21% for LDL (Table 3). These estimates are population-specific, but comparable to what has been reported before using SNP data in large European ancestry populations [6, 8].

**Table 3:** Heritability estimates for each lipid trait

Trait	Genetic Variance explained^a^ [Lower 95CI, Upper 95CI]	Pvalue (Genetic variance)	Variance explained by sampling unit (block group)	Variance explained by household	Variance explained by environment
Total cholesterol	0.22 [0.15, 0.29]	<0.001	0.002	0.047	0.731
Triglycerides	0.24 [0.18, 0.31]	<0.001	0.003	0.027	0.73
HDL-cholesterol	0.24 [0.17, 0.3]	<0.001	0.012	0.056	0.694
LDL-cholesterol	0.21 [0.14, 0.27]	<0.001	0.001	0.058	0.735

Lower 95CI: lower 95% confidence interval, Upper 95CI: upper 95% confidence interval

^a^ Genetic Variance: is the proportion of variance accounted for by relatedness

### Generalization

We tested all known signals that had been previously associated with a given lipid trait for generalization in the HCHS/SOL using directional r-values. For total cholesterol, we tested 121 previously published variants in 74 regions. Of these, 36 regions generalized to the HCHS/SOL (Additional file 1: Tables S5, S9), two of which (*HLA* and *PLEC1* regions) were based on secondary signals only. For LDL, we tested 128 published variants in 60 regions (Additional file 1: Tables S6, S9). Of these, 28 generalized to HCHS/SOL, one of which (*LDLRAP1*) was based on a secondary signal only. For HDL, we tested 139 published variants in 75 regions. Thirty-one of the 75 regions generalized to the HCHS/SOL (Additional file 1: Tables S7, S9), one of which (*LILRA3*) was based on secondary signals only. Finally, we tested 79 published triglycerides variants in 44 regions (Additional file 1: Tables S8, S9). Of these, 19 regions generalized to the HCHS/SOL, one of which was based on a secondary signal (*HLA*). In general, 50% of the tested SNPs generalized, overall and by ancestry, notably 11 out of the 12 SNPs previously identified in studies of Hispanic/Latino ancestry replicated in HCHS/SOL.

**Fig. 2:**
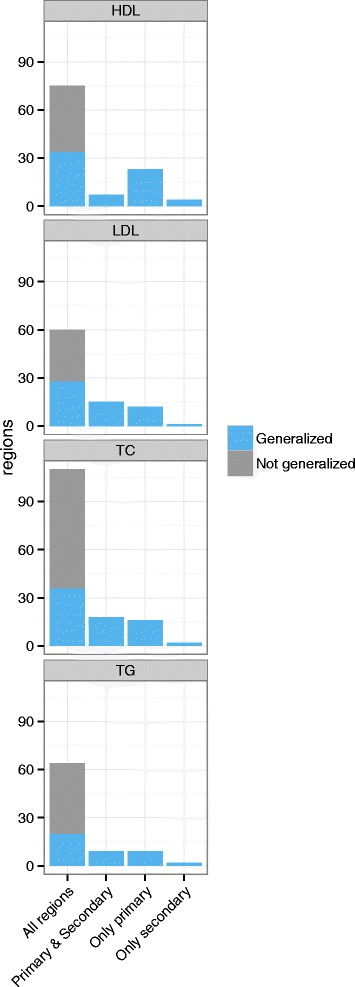
The number of generalized and non-generalized regions per trait. The number of generalized regions are divided according to the number of region generalizations that are due solely to generalization of the primary SNP (while other secondary SNPs did not generalize): “Only primary”; solely due to generalizations of a (at least one) secondary SNP (while the primary SNP did not generalize): “Only secondary”; and the number of regions in which both the primary SNP generalized, and at least one secondary SNP: “Primary & Secondary”. Those that generalized are shown in blue and those that did not generalize are shown in grey

We defined a region as generalized if at least one of the variants in the region generalized to HCHS/SOL. Figure 2 shows the number of generalized and non-generalized regions per trait. A region could have generalized because the primary SNP generalized, because a secondary SNP generalized, or because both did. There were nine regions in which the primary SNP did not generalize, while a secondary SNP did generalize (4 regions for HDL, 2 regions for total cholesterol, 1 region for LDL, 2 regions for triglycerides). In 49 generalized regions, only the primary SNP generalized, but no additional secondary SNP generalized. In the 48 remaining generalized regions, both the primary SNP and at least one secondary SNP generalized. Across all associations (regardless of regions), 43% of the primary SNPs (109 of 253 SNPs) generalized, and 59% of the secondary SNPs (123 of 209 total SNPs) generalized.

Finally, for each trait, to assess the possibility that additional previously reported associations exists in the HCHS/SOL, but could not be generalized due to lack of power, we calculated a score defined by the sum of all trait-increasing alleles of all the non-generalized SNPs. The results were highly significant: the *p*-value for genetic scores by trait were: HDL p-value 8.6 × 10^−26^, LDL p-value 1.4 × 10^−29^, triglycerides p-value 6.9 × 10^−41^, and total cholesterol p-value 3.5 × 10^−15^. These results suggest that some of the non-generalized SNPs are truly associated with their respective trait in the HCHS/SOL, but did not generalize individually due to lack of power.

## Discussion

We performed a GWAS of four lipid traits in a sample of approximately 12,800 Hispanic/Latino participants of HCHS/SOL. Of eight potentially novel loci identified from conditioning on known loci in the literature, we were unable to replicate any SNPs in several diverse replication cohorts. We demonstrated that >50% of the previously identified GWAS loci for lipid traits generalized to HCHS/SOL, which has important implications for further study of lipids genetics in Hispanics/Latinos.

We failed to replicate the eight potentially novel signals, possibly because of the very low minor allele frequencies, which ranged from 0.2% to 0.7% in the HCHS/SOL sample. At these frequencies and the identified effect sizes, our replication samples were not powered at 80% to replicate these effects. Studies in larger samples of Hispanic/Latino or diverse ancestry participants are needed to further investigate these low frequency variants. The signal that came closest to replicating was a signal in *DAGLB (*rs77071750 with MAF = 0.003) associated with HDL. rs77071750 lies about 130 kb from the primary signal, rs702485, and is monomorphic in populations of European ancestry but has a frequency of about 4% in populations of African ancestry. The SNP rs77071750 is intronic to *GRID2IP*, which encodes the glutamate receptor, ionotropic, delta 2 (Grid2) interacting protein that links GRID2 with actin cytoskeleton signaling molecules. As for the other potentially novel SNPs, while rs184637772 did not replicate, it has some interesting biology. It is intronic to *APOC3* has histone marks H3K4me3, H3K9ac indicative of promoter activity in liver and intestine. Other potentially novel SNPs appear less interesting.

Generalization analysis revealed that >50% of previously established lipid variants identified in GWAS of European-, Hispanic/Latino-, East Asian-, or African-descent populations generalized to HCHS/SOL based on an r-value <0.05, which accounts for agreement in the direction of effect. This fraction is much greater than expected by chance for each trait (binomial test for consistent direction of effect and an r-value <0.05 reached *p*-values < 6.5 × 10^−31^ for each trait). We might expect that loci that are directionally consistent and generalize are also likely to be functionally involved in lipid biology across diverse population groups. On the other hand, Hispanic/Latino descent populations have a fair amount of European ancestry and this could also be a reason for generalization. Failure to generalize can occur because the power for discovery in the HCHS/SOL was low, due to either low MAF or differences in LD across populations, chip coverage of the relevant locus, or because the originally published variant was a false positive.

We note that of the 12 SNPs (11 for HDL and 1 for triglycerides) that were previously identified in Hispanic/Latino samples, 11 generalized to HCHS/SOL. Ten of the 12 SNPs identified in Hispanic/Latino samples lie in previously established 1 Mb regions identified in Europeans, while two SNPs (both associated with HDL), rs148533712 (in *RORA*) and rs78557978 (near *UGT8*), lie in 1 Mb regions first identified in Hispanics/Latino samples. However, neither rs148533712 nor rs78557978 generalized to HCHS/SOL. Of the 10 SNPs that are in previously identified 1 Mb regions all of which generalized to HCHS/SOL), five associated with HDL are in LD (R^2^ > 0.5) with another previously identified European SNP or signal (rs2278426 in *LOC55908* and the *ANGPTL8* known region, rs1532624 near *CETP,* rs261334 and rs1077835 in/near *LIPC*, and rs4149310 in *ABCA1*). Except for rs4149310, all have no remaining signal (*p*-values > 0.05) after conditional analyses. However, rs4149310 still has some signal remaining, specifically an unconditioned p-value =1.52 × 10^−12^ that becomes p-value = 5.6 × 10^−08^ after conditioning on all known SNPs. An additional SNP associated with HDL, rs2472386 in the *ABCA1* region, identified in a different Hispanic sample than rs4149310, is in moderate LD with rs4149310 (r^2^ = 0.6) and in low LD with a nearby European identified SNP, rs2853579 (r^2^ = 0.3). However, between these 3 SNPs (i.e. rs2853579, rs4149310, and rs2472386) there is a distinct haplotype (TTG) that is identified in the 1000 genomes phase 3 AMR populations at 1% frequency and not found in the EUR populations. Two SNPs associated with HDL, rs9282541 a missense variant in the *ABCA1* gene, and rs11216230 an intronic variant in *SIK3* gene (also in the *APOA5/APOA1* known region) are Hispanic-specific signals and independent of all other known signals in their respective regions [12, 13]. We replicate these two signals for the first time in another Hispanic sample. Within the *APOA5/APOA1* region for the HDL, rs11216230 is the only SNP in a secondary signal that generalized. The other 4 SNPs in secondary signals did not replicate. Finally, a Hispanic-specific signal in the *CLIP2* region, rs8102280, an intronic variant in the *MAU2* gene, generalized to the HCHS/SOL sample. This signal has been replicated previously [9].

## Conclusions

In summary, we did not identify any novel lipid-associated loci, but did demonstrate that greater than half of previously identified GWAS loci for lipid traits generalized to HCHS/SOL*.* These results suggest that the genetic architecture of lipid levels includes several loci that are shared across different population groups. It is also possible that the generalization of European signals is due to the large fractions of European ancestry in Hispanics. Larger sample sizes are required for further investigations of potentially novel loci in Hispanic populations.

## Additional files

Additional file 1: Supplementary Tables. (ODS 129 kb)

Additional file 2: Supplementary Figures. (DOCX 112 kb)

## References

[1] Kannel WB, Dawber TR, Kagan A, Revotskie N, Stokes J (1961). Factors of risk in the development of coronary heart disease--six year follow-up experience. The Framingham study. Ann Intern Med.

[2] Rodriguez, C. J., M. L. Daviglus, K. Swett, H. M. Gonz??lez, L. C. Gallo, S. Wassertheil-Smoller, A. L. Giachello, Y. Teng, N. Schneiderman, G. A. Talavera, and R. C. Kaplan. 2014. Dyslipidemia patterns among Hispanics/Latinos of diverse background in the United States. Am J Med 127: 1186–1194.e1. 10.1016/j.amjmed.2014.07.026 PMC4551715 25195188

[3] Toth PP, Potter D, Ming EE (2012). Prevalence of lipid abnormalities in the United States: the National Health and nutrition examination survey 2003-2006. J Clin Lipidol.

[4] Third Report of the National Cholesterol Education Program (NCEP) (2002). Expert panel on detection, evaluation, and treatment of high blood cholesterol in adults (adult treatment panel III) final report. Circulation.

[5] Fenger M (2007). Heritability and genetics of lipid metabolism. Futur Lipidol.

[6] Willer CJ, Schmidt EM, Sengupta S, Peloso GM, Gustafsson S, Kanoni S, Ganna A, Chen J, Buchkovich ML, Mora S, Beckmann JS, Bragg-Gresham JL, Chang H-Y, Demirkan A, Den Hertog HM, Do R, Donnelly LA, Ehret GB, Esko T, Feitosa MF, Ferreira T, Fischer K, Fontanillas P, Fraser RM, Freitag DF, Gurdasani D, Heikkila K, Hypponen E, Isaacs A, Jackson AU, Johansson A, Johnson T, Kaakinen M, Kettunen J, Kleber ME, Li X, Luan J, Lyytikainen L-P, Magnusson PKE, Mangino M, Mihailov E, Montasser ME, Muller-Nurasyid M, Nolte IM, O’Connell JR, Palmer CD, Perola M, Petersen A-K, Sanna S, Saxena R (2013). Discovery and refinement of loci associated with lipid levels. Nat Genet.

[7] Teslovich TM, Musunuru K, Smith AV, Edmondson AC, Stylianou IM, Koseki M, Pirruccello JP, Ripatti S, Chasman DI, Willer CJ, Johansen CT, Fouchier SW, Isaacs A, Peloso GM, Barbalic M, Ricketts SL, Bis JC, Aulchenko YS, Thorleifsson G, Feitosa MF, Chambers J, Orho-Melander M, Melander O, Johnson T, Li X, Guo X, Li M, Shin Cho Y, Jin Go M, Jin Kim Y, Lee J-Y, Park T, Kim K, Sim X, Twee-Hee Ong R, Croteau-Chonka DC, Lange LA, Smith JD, Song K, Hua Zhao J, Yuan X, Luan J, Lamina C, Ziegler A, Zhang W, Zee RYL, Wright AF, Witteman JCM, Wilson JF, Willemsen G, et al. Biological, clinical and population relevance of 95 loci for blood lipids. Nature. 2010;466:707–13. 10.1038/nature09270 PMC3039276 20686565

[8] Tada H, Won H-H, Melander O, Yang J, Peloso GM, Kathiresan S (2014). Multiple associated variants increase the heritability explained for plasma lipids and coronary artery disease. Circ Cardiovasc Genet.

[9] Below JE, Parra EJ, Gamazon ER, Torres J, Krithika S, Candille S, Lu Y, Manichakul A, Peralta-Romero J, Duan Q, Li Y, Morris AP, Gottesman O, Bottinger E, Wang X-Q, Taylor KD, Ida Chen Y-D, Rotter JI, Rich SS, Loos RJF, Tang H, Cox NJ, Cruz M, Hanis CL, Valladares-Salgado A (2016). Meta-analysis of lipid-traits in Hispanics identifies novel loci, population-specific effects, and tissue-specific enrichment of eQTLs. Sci Rep.

[10] Dumitrescu L, Carty CL, Taylor K, Schumacher FR, Hindorff LA, Ambite JL, Anderson G, Best LG, Brown-Gentry K, Bůžková P, Carlson CS, Cochran B, Cole SA, Devereux RB, Duggan D, Eaton CB, Fornage M, Franceschini N, Haessler J, Howard BV, Johnson KC, Laston S, Kolonel LN, Lee ET, MacCluer JW, Manolio TA, Pendergrass SA, Quibrera M, Shohet RV, Wilkens LR, Haiman CA, Le Marchand L, Buyske S, Kooperberg C, North KE, Crawford DC (2011). Genetic determinants of lipid traits in diverse populations from the population architecture using genomics and epidemiology (PAGE) study. PLoS Genet.

[11] Elbers CC, Guo Y, Tragante V, van Iperen EPA, Lanktree MB, Castillo BA, Chen F, Yanek LR, Wojczynski MK, Li YR, Ferwerda B, Ballantyne CM, Buxbaum SG, Chen YDI, Chen WM, Cupples LA, Cushman M, Duan Y, Duggan D, Evans MK, Fernandes JK, Fornage M, Garcia M, Garvey WT, Glazer N, Gomez F, Harris TB, Halder I, Howard VJ, Keller MF, Kamboh MI, Kooperberg C, Kritchevsky SB, LaCroix A, Liu K, Liu Y, Musunuru K, Newman AB, Onland-Moret NC, Ordovas J, Peter I, Post W, Redline S, Reis SE, Saxena R, Schreiner PJ, Volcik KA, Wang X, Yusuf S, Zonderland AB (2012). Gene-centric meta-analysis of lipid traits in African, east Asian and Hispanic populations. PLoS One.

[12] Weissglas-Volkov D, Aguilar-Salinas CA, Nikkola E, Deere KA, Cruz-Bautista I, Arellano-Campos O, Munoz-Hernandez LL, Gomez-Munguia L, Ordonez-Sanchez ML, Reddy PMVL, Lusis AJ, Matikainen N, Taskinen M-R, Riba L, Cantor RM, Sinsheimer JS, Tusie-Luna T, Pajukanta P (2013). Genomic study in Mexicans identifies a new locus for triglycerides and refines European lipid loci. J Med Genet.

[13] Ko A, Cantor RM, Weissglas-Volkov D, Nikkola E, Reddy PMVL, Sinsheimer JS, Pasaniuc B, Brown R, Alvarez M, Rodriguez A, Rodriguez-Guillen R, Bautista IC, Arellano-Campos O, Muñoz-Hernández LL, Salomaa V, Kaprio J, Jula A, Jauhiainen M, Heliövaara M, Raitakari O, Lehtimäki T, Eriksson JG, Perola M, Lohmueller KE, Matikainen N, Taskinen M-R, Rodriguez-Torres M, Riba L, Tusie-Luna T, Aguilar-Salinas CA, Pajukanta P (2014). Amerindian-specific regions under positive selection harbour new lipid variants in Latinos. Nat Commun.

[14] Wu Y, Waite LL, Jackson AU, Sheu WH-H, Buyske S, Absher D, Arnett DK, Boerwinkle E, Bonnycastle LL, Carty CL, Cheng I, Cochran B, Croteau-Chonka DC, Dumitrescu L, Eaton CB, Franceschini N, Guo X, Henderson BE, Hindorff LA, Kim E, Kinnunen L, Komulainen P, Lee W-J, Le Marchand L, Lin Y, Lindstrom J, Lingaas-Holmen O, Mitchell SL, Narisu N, Robinson JG, Schumacher F, Stancakova A, Sundvall J, Sung Y-J, Swift AJ, Wang W-C, Wilkens L, Wilsgaard T, Young AM, Adair LS, Ballantyne CM, Buzkova P, Chakravarti A, Collins FS, Duggan D, Feranil AB, Ho L-T, Hung Y-J, Hunt SC, Hveem K (2013). Trans-ethnic fine-mapping of lipid loci identifies population-specific signals and allelic heterogeneity that increases the trait variance explained. PLoS Genet.

[15] Zubair N, Graff M, Ambite JL, Bush WS, Kichaev G, Lu Y, Manichaikul A, Sheu WH-H, Absher D, Assimes TL, Bielinski SJ, Bottinger EP, Buzkova P, Chuang L-M, Chung R-H, Cochran B, Dumitrescu L, Gottesman O, Haessler JW, Haiman C, Heiss G, Hsiung CA, Hung Y-J, Hwu C-M, Juang J-MJ, Le Marchand L, Lee I-T, Lee W-J, Lin L-A, Lin D, Lin S-Y, Mackey RH, Martin LW, Pasaniuc B, Peters U, Predazzi I, Quertermous T, Reiner AP, Robinson J, Rotter JI, Ryckman KK, Schreiner PJ, Stahl E, Tao R, Tsai MY, Waite LL, Wang T-D, Buyske S, Chen Y-DI, Cheng I (2016). Fine-mapping of lipid regions in global populations discovers ethnic-specific signals and refines previously identified lipid loci. Hum Mol Genet.

[16] Sorlie PD, Aviles-Santa LM, Wassertheil-Smoller S, Kaplan RC, Daviglus ML, Giachello AL, Schneiderman N, Raij L, Talavera G, Allison M, LaVange L, Chambless LE, Heiss G (2010). Design and implementation of the Hispanic community health study/study of Latinos. Ann Epidemiol.

[17] LaVange, L. M., W. D. Kalsbeek, P. D. Sorlie, L. M. Avil??s-Santa, R. C. Kaplan, J. Barnhart, K. Liu, A. Giachello, D. J. Lee, J. Ryan, M. H. Criqui, and J. P. Elder. 2010. Sample design and cohort selection in the Hispanic community health study/study of Latinos. Ann Epidemiol 20: 642–649. 10.1016/j.annepidem.2010.05.006 PMC2921622 20609344

[18] Conomos MP, Laurie CA, Stilp AM, Gogarten SM, McHugh CP, Nelson SC, Sofer T, Fernández-Rhodes L, Justice AE, Graff M, Young KL, Seyerle AA, Avery CL, Taylor KD, Rotter JI, Talavera GA, Daviglus ML, Wassertheil-Smoller S, Schneiderman N, Heiss G, Kaplan RC, Franceschini N, Reiner AP, Shaffer JR, Barr RG, Kerr KF, Browning SR, Browning BL, Weir BS, Avilés-Santa ML, Papanicolaou GJ, Lumley T, Szpiro AA, North KE, Rice K, Thornton TA, Laurie CC. Genetic diversity and association studies in US Hispanic/Latino populations: applications in the Hispanic community health study/study of Latinos. Am J Hum Genet. 2016;98:165–84. 10.1016/j.ajhg.2015.12.001 PMC4716704 26748518

[19] Daviglus ML, Talavera GAG, Avilés-Santa ML, Allison M, Cai J, Criqui MH, Gellman M, Giachello AL, Gouskova N, Kaplan RC, LaVange L, Penedo F, Perreira K, Pirzada A, Schneiderman N, Wassertheil-Sctors and cardiovascular diseases among Hispanic/Latino individuals of diverse bmoller S, Sorlie PD, Stamler J. Prevalence of major cardiovascular risk faackgrounds in the United States. JAMA. 2012;308:1775–84. 10.1001/jama.2012.14517 PMC3777250 23117778

[20] Friedewald WT, Levy RI, Fredrickson DS. Estimation of the concentration of low-density lipoprotein cholesterol in plasma, without use of the preparative ultracentrifuge. Clin Chem. 1972;18:499–502. 4337382

[21] Wu J, Province MA, Coon H, Hunt SC, Eckfeldt JH, Arnett DK, Heiss G, Lewis CE, Ellison RC, Rao DC, Rice T, Kraja AT (2007). An investigation of the effects of lipid-lowering medications: genome-wide linkage analysis of lipids in the HyperGEN study. BMC Genet.

[22] Sweeney ME, Johnson RR (2007). Ezetimibe: an update on the mechanism of action, pharmacokinetics and recent clinical trials. Expert Opin Drug Metab Toxicol.

[23] Design of the Women’s Health Initiative clinical trial and observational study (1998). The Women’s health initiative study group. Control Clin Trials.

[24] Lorenzo C, Wagenknecht LE, D’Agostino RB, Rewers MJ, Karter AJ, Haffner SM (2010). Insulin resistance, beta-cell dysfunction, and conversion to type 2 diabetes in a multiethnic population: the insulin resistance atherosclerosis study. Diabetes Care.

[25] Henkin L, Bergman R, Bowden D, Ellsworth D, Haffner S, Langefeld C, Mitchell B, Norris J, Rewers M, Saad M, Stamm E, Wagenknecht L, Rich S (2003). Genetic epidemiology of insulin resistance and visceral adiposity the IRAS family study design and methods. Ann Epidemiol.

[26] Xiang, A., S. Azen, L. Raffel, S. Tan, L. Cheng, T. Diaz, J, E. Toscano, P. Henderson, H. Hodis, W. Hsueh, R. JI, and B. TA. 2001. Evidence for joint genetic control of insulin sensitivity and systolic blood pressure in Hispanic families with a hypertensive proband. Circulation 103: 78083. 10.1161/01.cir.103.1.78 11136689

[27] Goodarzi MO, Guo X, Taylor KD, Quiñones MJ, Samayoa C, Yang H, Saad MF, Palotie A, Krauss RM, Hsueh WA, Rotter JI (2003). Determination and use of haplotypes: ethnic comparison and association of the lipoprotein lipase gene and coronary artery disease in Mexican-Americans. Genet Med.

[28] Hill C, Gerardo D, James F, Tyroler HA, Chambless LE, Romm J, Disanto AR, Barr K, Bergsten J, Conrad J, Elliott R, Furr D, Hafer B, Haire A, Jensen J, Johnson P, Marlow J, Monger B, Mooney D, Posey D, Sofley C, Tatum C, Toledo A, Langford H, Asken B, Blackburn F, Bowman C, Feild L, Franklin R, Hathorn D, Howell R, Nelson M, Overman V, Oxner S, Pitts D, Shelton G, Edlavitch S, Cram K, Reed L, Murton G, Nabulsi A, Bowers M, Kuehl B, Hamele H, Christman C, Costa D, Har S, Markam T, Neuing J (1989). The atherosclerosis risk in communities (ARIC) study: design and objectives. The ARIC investigators. Am J Epidemiol.

[29] Sofer T, Shaffer JR, Graff M, Qi Q, Stilp AM, Gogarten SM, North KE, Isasi CR, Laurie CC, Szpiro AA (2016). Meta-analysis of genome-wide association studies with correlated individuals: application to the Hispanic community health study/study of Latinos (HCHS/SOL). Genet Epidemiol.

