# Supplementary figures and images for: Genetic architecture of lipid traits in the Hispanic community health study/study of Latinos
Source: Lipids Health Dis. 2017 Oct 12;16:200. doi: 10.1186/s12944-017-0591-6 (PMC5639746; doi:10.1186/s12944-017-0591-6)

Figure S2 Quantile-quantile plots for each lipid trait


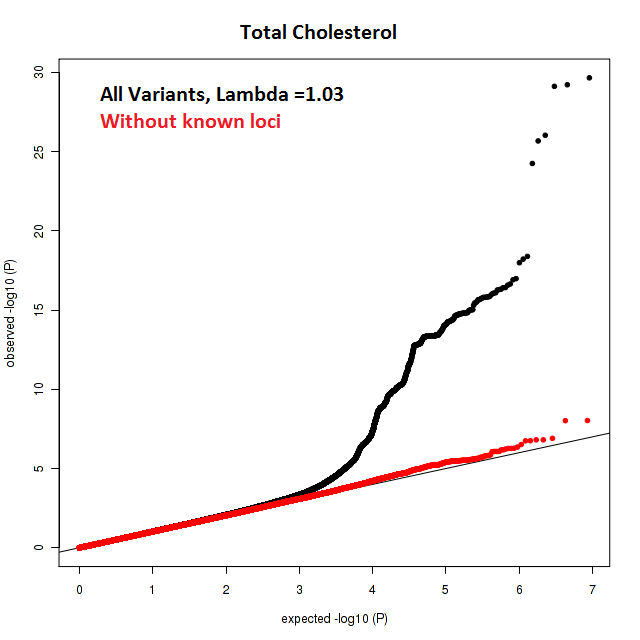

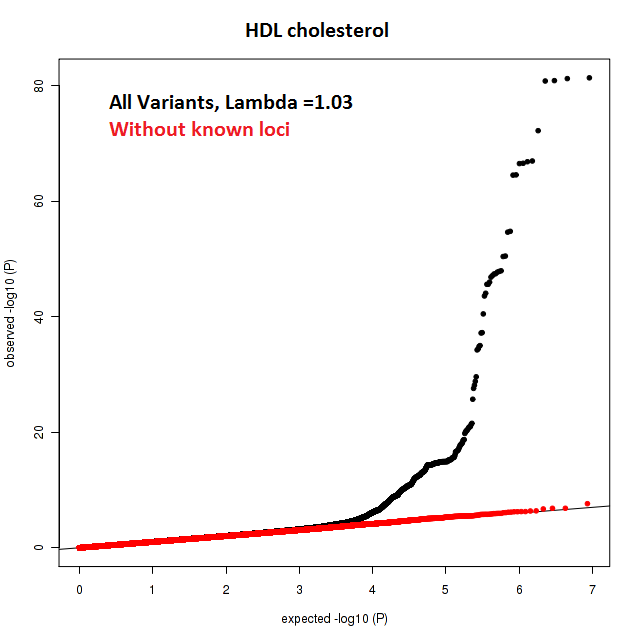


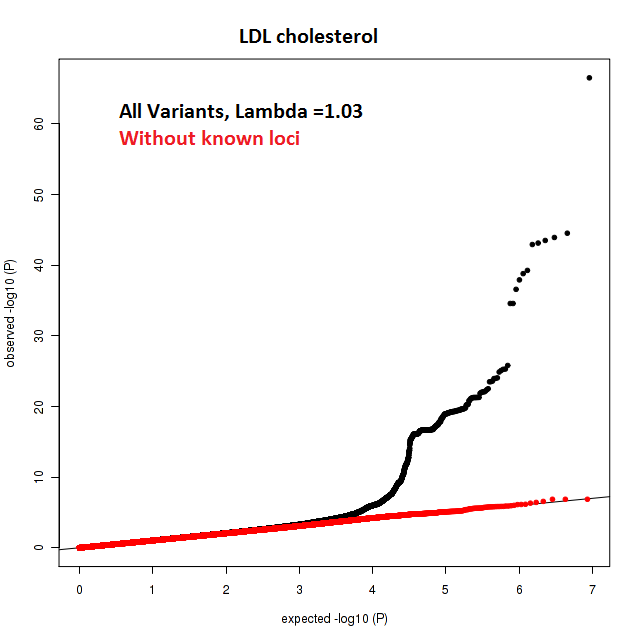

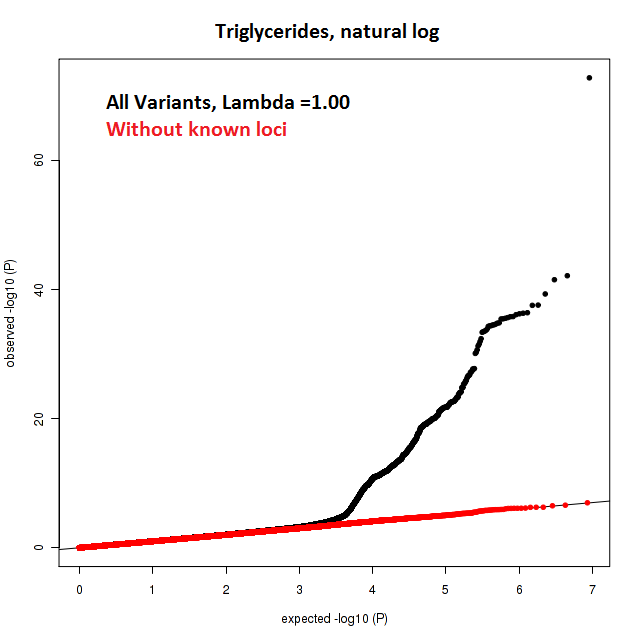

Supplement: Supplementary file 2 — Supplementary Figures. (DOCX 112 kb) [file 12944_2017_591_MOESM2_ESM.docx]
